# The role of mTOR-mediated signals during haemopoiesis and lineage commitment

**DOI:** 10.1042/BST20180141

**Published:** 2018-08-28

**Authors:** Natasha Malik, Owen J. Sansom, Alison M. Michie

**Affiliations:** 1 Institute of Cancer Sciences, College of Medicine, Veterinary and Life Sciences, University of Glasgow, Glasgow, U.K.; 2 Cancer Research UK Beatson Institute, Garscube Estate, Glasgow, U.K.

**Keywords:** haemopoiesis, intracellular signalling, mechanistic target of rapamycin

## Abstract

The serine/threonine protein kinase mechanistic target of rapamycin (mTOR) has been implicated in the regulation of an array of cellular functions including protein and lipid synthesis, proliferation, cell size and survival. Here, we describe the role of mTOR during haemopoiesis within the context of mTORC1 and mTORC2, the distinct complexes in which it functions. The use of conditional transgenic mouse models specifically targeting individual mTOR signalling components, together with selective inhibitors, have generated a significant body of research emphasising the critical roles played by mTOR, and individual mTOR complexes, in haemopoietic lineage commitment and development. This review will describe the profound role of mTOR in embryogenesis and haemopoiesis, underscoring the importance of mTORC1 at the early stages of haemopoietic cell development, through modulation of stem cell potentiation and self-renewal, and erythroid and B cell lineage commitment. Furthermore, the relatively discrete role of mTORC2 in haemopoiesis will be explored during T cell development and B cell maturation. Collectively, this review aims to highlight the functional diversity of mTOR signalling and underline the importance of this pathway in haemopoiesis.

## Introduction

Mechanistic target of rapamycin (mTOR) is a serine/threonine protein kinase which was initially identified due to its ability to be inhibited by rapamycin, an antifungal macrolide first characterised in *Streptomyces hygroscopicus*. Purification and identification of mTOR in mammals subsequently revealed that it regulates a plethora of biological functions, including protein and lipid synthesis, mitochondrial function, autophagy and cytoskeleton organisation, contributing towards proliferation and cell survival [1]. However, the mechanisms regulated by mTOR signalling in specific cell contexts are still not fully understood.

## The mTOR-mediated signalling pathway

**Figure 1.**
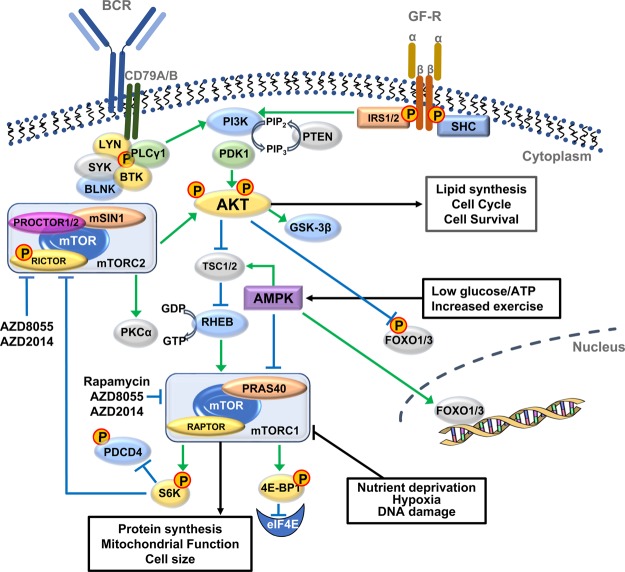
Diagram of the AKT/mTOR Signalling Pathway. Summary of downstream signalling from the BCR and growth factor receptors (GF-R) is shown. Activation of these receptors results in phosphorylation and activation of Akt, which leads to the activation of mTORC1 (PRAS40, RAPTOR), thereby initiating cell processes such as protein synthesis and proliferation. The downstream target of mTORC1, S6K negatively regulates mTORC2 (PROTOR1/2, mSIN1, RICTOR), which is responsible for the activation of Akt. This creates a negative feedback loop, which regulates this pathway. mTORC1 and mTORC2 share the subunits mTOR, GβL, DEPTOR and Tti1/Tel2 (not shown). Akt negatively phosphorylates FOXO1/3, which regulates the cell cycle. Kinases such as AMPK are activated in stress responses and inhibit the mTORC1 pathway. Allosteric inhibitors such as rapamycin and other rapalogs partially inhibit mTORC1 activity, whereas ATP competitive inhibitors such as AZD2014 are pan mTOR inhibitors. All inhibitory and non-inhibitory signalling is represented in blue and green, respectively. PLC, phospholipase C; BLNK, B cell linker; BTK, Bruton tyrosine kinase.

mTOR is activated by a variety of upstream mediators including growth factor (GF) receptors (e.g. insulin/insulin-like GF, tumour necrosis factor receptors), and nutrients such as glucose and amino acids (AAs). mTOR belongs to the phosphoinositide 3-kinase (PI3K)-related kinase (PIKK) family and forms two distinct complexes — mTORC1 and mTORC2. These complexes share the proteins mTOR, GβL, DEPTOR and Tti1/Tel2. The subunits which make the respective complexes unique are RAPTOR (rapamycin TOR sensitive) and PRAS40 contained within mTORC1, and RICTOR (rapamycin TOR insensitive), mSIN1 and PROCTOR1/2 specific to mTORC2 [1]. As suggested by the names, RAPTOR and RICTOR define the sensitivity of mTORC1 and mTORC2 to rapamycin [2]. Rapamycin interacts with FK506-binding proteins, such as FKBP12, with high affinity and the resultant complex interacts with mTOR in the context of mTORC1 to allosterically inhibit mTORC1 activity [3,4]. PI3K, an upstream mediator of mTOR, can be activated through GF receptor activation, leading to PI3K binding to insulin receptor substrate (IRS) proteins and conversion of phosphatidylinositol-4,5-phosphate (PIP_2_) into phosphatidylinositol-3,4,5-phosphate (PIP_3_) (Figure 1). Phosphatase and tensin homolog (PTEN), a tumour suppressor, reduces PIP_3_ accumulation, while PI3K promotes the activation of 3-phosphoinositide-dependent protein kinase 1 (PDK1), which then phosphorylates and activates AKT [5]. AKT then activates mTORC1 by inhibiting tuberous sclerosis complex (TSC)1/2. TSC proteins form a heterodimer and inhibit Ras homolog enriched in brain (RHEB), a positive regulator of mTORC1, which binds to mTORC1 causing conformational changes in the protein and activation [5].

### mTORC1 signalling

RAPTOR binds mTOR to enhance its activity, as indicated by the finding that RAPTOR inhibition, using RNAi, leads to decreased mTOR activity [6]. In a low-energy state (low ATP:AMP ratio), AMP-protein kinase (AMPK), a conserved energy sensor, is activated, leading to TSC2 phosphorylation and the subsequent inhibition of mTORC1 activity [7]. However, in a high-energy state, the mTORC1 pathway is activated, promoting protein synthesis, lipogenesis, and mitochondrial biogenesis and function [8–11]. Indeed, mTORC1 plays a pivotal role in mitochondrial oxidative function through regulation of the transcription factor yin-yang1 (YY1), which subsequently controls gene expression of mitochondrial transcriptional regulators including PGC-1α [10]. Furthermore, mTORC1 controls mitochondrial biogenesis and respiration through phosphorylation/inhibition of the eukaryotic initiation factor 4E (eIF-4E) binding proteins (4E-BPs) and regulation of the translation of nucleus-encoded mitochondrial-related mRNAs, which in turn increases ATP generation in the cell [11]. In the absence of mTORC1 activity, 4EBP1 is hypophosphorylated and interacts with the mRNA cap-binding protein eIF4E, inhibiting translation of cap-dependent proteins. Upon mTOR activation, hyperphosphorylation of 4E-BP1 releases eIF4E, enabling its association with eIF-4A (RNA helicase) and the scaffolding protein eIF-4G to form the eIF-4F complex (Figure 1). The mTORC1-eIF4E pathway is up-regulated in most cancers and thus represents an attractive therapeutic target [12].

mTORC1 also phosphorylates/activates S6 Kinase 1 (S6K1) at Thr^389^, which was initially thought to play a role in protein/ribosomal biogenesis by activating 40S ribosomal protein. However, it is now appreciated that S6K1 is important in many mechanisms, together with S6K2, including transcription, cell proliferation, apoptosis and potential mRNA splicing [13]. S6K phosphorylates programmed cell death 4 protein (PDCD4) at Ser^67^, targeting it for proteasomal degradation [14]. PDCD4 is a tumour suppressor responsible for inhibiting eIF4E. Moreover, a recent study in a colorectal cancer model shows that S6K phosphorylates and inhibits elongation factor-2 kinase (EF2K), in turn relieving EF2K inhibition of EF2 and thus elongation of nascent polypeptide chains [15]. S6K also plays a role in actin organisation by the direct binding to F-actin. A role in cytoskeletal rearrangement is also seen as S6K activates Rho family members regulating this — Cdc42 and Rac1 and their downstream target PAK1. As deletion of S6K leads to a decrease in activation of the Rho family members, cytoskeletal organisation and migration in ovarian cancer cells, S6K is a promising target for enabling reduction in tumour progression [16]. An additional target of S6K1 is RICTOR [17], which, when phosphorylated at Thr^1135^, leads to mTORC2 inhibition, establishing a negative feedback loop between mTORC1 and mTORC2 [18].

### mTORC2 signalling

AKT can be considered the hub of the PI3K pathway as its downstream signalling leads to mechanisms controlling a multitude of diverse functions within the cell. AKT is activated upon phosphorylation of two key sites, Thr^308^ by PDK1 and Ser^473^ by mTORC2, via GF receptor activation [19]. The majority of mTORC2 functions occur through AKT regulation, including activation of mTORC1, placing AKT both upstream of and downstream from mTOR regulation. During low-energy conditions, AMPK activation leads to an up-regulation of mTORC2 activity and modulation of downstream targets [20]. Such downstream targets include the Forkhead Box O (FOXO) family of transcription factors, which when phosphorylated by AKT leads to an inhibition of their function. FOXOs play an important role in the repression of cell proliferation and survival, but in certain cell contexts can also play a role in tumorigenesis [21]. FOXOs regulate apoptosis in distinct ways, repressing apoptosis through the down-regulation of the pro-apoptotic BCL2 family member Bim, or promoting apoptosis through transcriptional up-regulation of the FAS ligand [22,23]. mTORC2-FOXO1 signalling also regulates innate immune responses as RICTOR deletion leads to attenuated AKT signalling, thereby increasing nuclear FOXO1, resulting in hyper-inflammatory responses via toll-like receptor 4 (TLR4) [24]. mTORC2 can localise at the mitochondria-associated endoplasmic reticulum membranes (MAMs) in a GF-dependent manner, and *RICTOR* deletion disrupts AKT-dependent phosphorylation of mitochondria-associated proteins. These events lead to a reduction in mitochondrial function, increasing mitochondrial membrane potential and affecting energy metabolism and cell survival, thereby demonstrating a vital role of mTORC2 signalling in mitochondrial physiology [25]. The importance of mTORC2 in AKT activation was highlighted by a recent study demonstrating that deletion of the AKT-binding site within the mTORC2 component mSIN1 greatly reduced AKT^S473^ phosphorylation, rendering it unable to phosphorylate FOXO1/3a, while other targets such as glycogen synthase kinase 3 (GSK3) and mTORC1 were unaffected [26,27]. These findings suggest that mTORC2 activation is important for AKT-mediated cell survival mechanisms, but not for mTORC1 mechanisms.

Additional targets of mTORC2 include protein kinase C-alpha (PKCα) as mTORC2 inactivation reduced PKCα phosphorylation [28], which is responsible for functions including cell proliferation, differentiation, motility, apoptosis and inflammation [29]. mTORC2 also regulates growth and ion transport by phosphorylating the hydrophobic motif of serum and glucocorticoid-induced protein kinase 1 (SGK1) [30]. SGK1 inhibition induces autophagy, apoptosis and cell cycle arrest in the G_2_/M phase in prostate cancer cell lines, at least in part through an mTOR-FOXO3a-mediated pathway [31]. SGK1 also regulates T_H_2 differentiation and negatively regulates interferon gamma (IFNγ) production, thereby highlighting the importance of mTORC2 in T cell effector function [32]. mTORC2 has also shown to play a role in cytoskeletal organisation by activating RhoA GTPases [33].

## mTOR in embryogenesis

The mTOR complexes are essential for cell survival and growth, and studies generating knockout (KO) mice established that mTOR kinase and individual complexes mTORC1/2 were essential for normal embryogenesis [34,35]. A homozygous KO of *mTOR* (*mTOR*^−/−^) resulted in the death of mouse embryos soon after implantation (E5.5–6.5). Despite normal blastocyst development, the embryo did not develop further due to limited proliferation and survival signalling. Nevertheless, *mTOR*^+/−^ mice developed fertile and normal embryos. Similarly, *Raptor*^−/−^ embryos die during early development (E7), whereas the *Rictor*^−/−^ mice survived slightly longer (E10.5) [35]. These studies indicate mTOR function is mediated mainly through mTORC1 during early embryogenesis, but both mTORC1/2 play critical roles.

## Role of mTOR signalling in haemopoiesis

Haemopoiesis initially occurs in the yolk sac and in two waves, the first of which is known as ‘primitive’ haemopoiesis. At this stage *de novo* haemangioblasts are generated and produce large quantities of erythrocytes to promote increased oxygenation, accommodating rapid growth. During the second wave of haemopoiesis or definitive haemopoiesis, haemopoietic stem cells (HSCs) appear in the aorta–gonad–mesonephros region around E10 [36]. From E11, HSCs migrate to and colonise the foetal liver (FL) and subsequently the bone marrow (BM) with waves of repopulating HSCs that provide a continuous source of mature haemopoietic lineage cells during the adult lifespan. The nature of the HSCs differ depending on the micro-environmental niche, with HSCs in the BM being more quiescent than those in the FL [37,38]. HSC differentiation into multipotent progenitor (MPP) cells occurs mainly in the FL prior to migration into specific haemopoietic organs, such as the thymus, for further lineage differentiation. MPPs give rise to oligopotent common myeloid or lymphoid progenitors (CMPs or CLPs). CMPs further give rise to megakaryocyte-erythroid progenitors and granulocyte–macrophage progenitors, while CLPs give rise to lymphoid lineage cells [39]. Targeted deletion of mTORC1 and/or mTORC2 in mouse models demonstrate a critical role for the mTOR pathway in haemopoiesis, and highlight the importance of the individual mTOR-containing complexes at specific stages of HSC homeostasis and haemopoietic lineage commitment and maturation, as discussed below.

### Haemopoietic stem cells

Conditional knockout (cKO) mouse models of PTEN and TSC1, upstream negative regulators of mTORC1 in HSCs, revealed an increase in short-term HSC cycling and a concomitant decline in long-term HSC (LT-HSC) quiescence and self-renewal through constitutive activation of mTORC1 [40–42]. TSC1^−/−^ in HSCs led to an elevation in mitochondrial biogenesis, resulting in increased reactive oxygen species (ROS) production, driving HSCs from quiescence to rapid cell cycling, thereby reducing their self-renewal capacity [43]. These studies identify the role of mTOR in regulating HSC cycling through modulation of ROS levels. Interestingly, similar findings were reported in *mTOR* cKO mice, in which BrdU labelling revealed rapid cell cycling of HSCs leading to a loss of quiescence and defective HSC engraftment and repopulation upon transplantation into NSG mice [44]. Recent studies establish cross-talk between the ERK and mTOR signalling pathways, with ERK activity regulating mTORC1 activation, thus limiting its strength to promote HSC cycling in favour of quiescence. Indeed, HSCs derived from MEK1 cKO mice exhibit exhaustion due to increased mTORC1-mediated ROS production, resulting in increased mitochondrial damage [45]. Collectively, these studies identify the importance of precise mTOR regulation during HSC maintenance and haemopoiesis.

The Wnt-signalling pathway can activate the mTOR pathway by inhibiting GSK3-mediated phosphorylation of TSC2 independently of β-catenin transcription. GSK3 inhibits mTOR activation by phosphorylating TSC2 in a manner co-ordinated by AMPK [46]. Knockdown (KD) of *Gsk3* initially resulted in an increase in Lin^−^Sca1^+^cKit^+^ (LSK) populations due to an activation of both Wnt-signalling (β-catenin-dependent) and mTOR pathways. However, long-term disruption of GSK3 expression/activity led to a depletion of HSC populations due to mTOR activation, which could be reversed through modulation of mTORC1 and β-catenin [47]. Inhibition of the mTOR pathway together with activation of the Wnt-β-catenin pathway led to increased LT-HSC number and the potential to culture HSCs *ex vivo* in a cytokine-free environment, highlighting the importance of appropriate regulation of the Wnt and mTOR signalling pathways in stem cell renewal [48].

Reduction in signalling downstream of mTOR enhances HSC self-renewal and repopulating properties: mice lacking S6K1 [49] or mice treated with rapamycin [50] exhibit an increased life- and health span compared with controls due to an increase in repopulating LT-HSCs. Collectively, these findings suggest that the loss in mTOR function during haemopoiesis primarily represents a loss of mTORC1 activity, with mTORC2 not playing a key role in HSCs.

### Haemopoietic stem/progenitor cell

Assessing the role of mTOR on haemopoietic lineage commitment using an *mTOR* cKO model in adult mice revealed significant aberrations in the development of haemopoietic lineage populations, resulting in a reduction in splenic weight and size. Closer analysis revealed that mTOR disruption led to pancytopenia, including a block in erythrocyte development at the pro-erythroblast stage, and anaemia [44]. The decline in haemopoietic lineage commitment was accompanied by increased apoptosis and decreased Mcl-1 expression. Within BM haemopoietic progenitor populations, there was a skew towards CMPs and a decrease in CLPs in *mTOR*-deficient mice. While there was an increase in the LSK population, the colony-forming ability of these LSKs was impaired. There was also an attenuation in S6K and 4E-BP1 phosphorylation/activation and increased phosphorylation of AKT, implicating an aberration in the S6K-mediated negative feedback loop regulation of mTORC2 activity [16,44].

The mTORC1 cKO model (*Raptor-Mx1*-cre) exhibited an increased LSK population in the spleen in addition to the BM, indicative of extramedullary haemopoiesis. The LSKs were arrested at the G_1_ phase of the cell cycle compared with controls, suggesting a reduction in cell division [51]. Metabolite analysis of LSKs revealed an increase in intermediates used in lipid metabolism, and in AMP and NADP involved in redox homeostasis, and a decrease in nitrogen metabolism [51]. LSK^−^CD48^−^CD150^+^ cells derived from the *Raptor*^−/−^ BM failed to engraft into recipient mice. While these cells were able to home to the BM, they localised further from osteoblast cells, indicating a role for mTORC1 in the integration of niche signals. *Raptor*^−/−^ HSCs also possessed regenerative and self-renewal aberrations compared with controls and those cells that ‘escaped deletion’. A compound *Rictor*/*Raptor* cKO in adult mice exhibited similar results as *Raptor*^−/−^ mice in haemopoiesis; however, this mouse model did not develop BM failure and retained the deletion of alleles of *Raptor* and *Rictor* for almost one year after KO induction [51]. These results suggest that the mTOR pathway is not essential for survival and haemopoietic maintenance in adult mice but is essential for haemopoiesis initiation during embryonic development.

Genetic targeting studies to ablate mTORC2 function in haemopoiesis revealed a more subtle role compared with mTORC1. Studies in *Rictor* cKO (*Rictor-Mx1*-cre) mice indicated that mTORC2 does not play a significant role in HSCs and progenitor populations [51,52]. However, Magee et al. elegantly demonstrated that after PTEN deletion, which activates the mTOR-signalling pathway, deletion of *Rictor* abrogates leukaemogenesis and HSC depletion in adult, but not neonatal, mice [53], highlighting that the PTEN-mTORC2 signalling axis has a role in activating these processes in a temporally dependent manner.

### Red blood cells

The mTOR cKO model revealed a block in erythropoiesis at the pro-erythroblast stage, highlighting the importance of mTOR signalling in RBC (red blood cell) development [44]. Interestingly, results from this model are similar to that observed in the *Tsc1* cKO mouse, which exhibited a reduction in erythrocytes in the BM [42], through activation of mTORC1-mediated signalling. These studies indicate that a complex regulation of mTOR signalling is required for appropriate RBC development. Analysis of the importance of mTOR function in erythropoiesis revealed that loss of FOXO3 in erythroblasts results in an overactivation of the mTOR pathway, thereby compromising erythroid maturation [54]. FOXO3 regulates GATA-1 expression and represses Exosc8 expression, which are both involved in erythroid maturation [55]. Additionally, ectopic expression of microRNA9 disrupts erythropoiesis via the suppression of FOXO3-mediated pathways, causing an increase in ROS due to the down-regulation in ROS-scavenging enzymes [56].

Recent studies show that mTORC1 plays a critical role in RBC commitment, growth, proliferation and homeostasis. Knight et al. [57] demonstrated that mTORC1 activity is regulated by dietary iron, and a loss or overexpression of mTORC1 in HSCs leads to microcytic or macrocytic anaemia, respectively, with a loss of proliferation in RBC progenitors. Furthermore, treatment of mice with the ATP competitive mTOR inhibitor MLN0128, and subsequently with phenylhydrazine to induce haemolysis, was shown to be lethal, demonstrating the reliance of the mTOR pathway in RBC development [57]. Zhang et al. have shown that the heme-regulated eIF2α kinase (HRI)-activating transcription factor 4 (ATF4) pathway, which regulates heme uptake for haemoglobin production and stress response genes, suppresses mTORC1 activity in iron deficiency anaemia. The HRI-ATF4 pathway promoted RBC progenitor differentiation, and pharmacological inhibition of mTORC1 rescued RBC counts and haemoglobin content in the blood [58]. mTOR also plays a role in micro-environmental homeostasis associated with RBC development, through regulation of neutral essential AA (NEAA) uptake into cells during erythropoiesis for haemoglobin production. mTORC1/4EBP1 signalling regulates *Lat3*, a transporter of NEAAs in RBCs [59].

### Myeloid lineage cells

cKOs of *Mtor* or *Raptor* in HSCs lead to a significant accumulation of the CD11b^+^Gr1^−^ population [60]. *mTOR* cKO mice on a SCID background exhibited reduced monocyte/macrophage populations in *in vitro* and *in vivo* assays. However, removal of mTOR expression specifically in myeloid cells (*Mtor*-Lyzs-cre) revealed normal levels of monocyte/macrophage populations, suggesting that mTOR plays a role during lineage commitment, but not during survival and maturation [61]. Following mTOR deficiency, a decrease in expression of the M-CSF receptor, CD115 was noted, which may result in decreased monocyte/macrophage populations due to overactive STAT5 and down-regulation of IRF8 [61]. M-CSF promotes mTORC1 activation, which further promotes CD115 expression, and expression of PU.1 and IRF8 to promote myelopoiesis. In the absence of mTORC1 activity there is a block in glucose uptake and lipid metabolism, thereby abrogating myeloid differentiation and generating an impaired immune response to bacterial infection [62]. Constitutive activation of mTORC1 in *TSC* KO BM-derived macrophages (BMDMs) attenuated AKT signalling through the negative feedback loop via mTORC2, leading to a defect in IL4-induced M2 polarisation. These BMDMs produce more pro-inflammatory responses compared with controls, suggesting an important role of mTORC1 in the regulation of inflammation [63]. Additionally, it has recently been shown that *Raptor* cKO, but not granulocyte-specific KO (*Raptor*-lyz2-cre) mice exhibit a significant increase and accumulation of innate myelo-lymphoblastoid effector cells (IMLECs). This suggests that IMLEC accumulation driven by *Raptor* deficiency occurs earlier in development, caused by reduced expression of *Myb* in CMPs [64].

A myeloid lineage-specific KO model of *Rictor* (lysM-cre) revealed a significant decrease in monocytes, while the neutrophil population was unaffected. BM monocytes and peritoneal macrophages displayed decreased proliferation and increased susceptibility to pro-apoptotic stimuli. Furthermore, stimulation of TLR4 on *Rictor*^−/−^ macrophages with LPS potentiated a pro-inflammatory response, with cells skewing towards an M1 phenotype and down-regulating IL10 expression, suggesting that mTORC2 signalling is a negative regulator of TLR signalling in macrophages [65,66]. Interestingly, the inflammatory response observed in *Rictor*^−/−^ cells was reversed by the inactivation of *Raptor*, indicating that mTORC1 regulates inflammatory responses in macrophages [65].

Mice with reduced/absent mTOR activity/expression also exhibit a reduction in neutrophils in peripheral blood [44,67]. Neutrophils release antimicrobial cargos, which are carried on chromatin fibres called neutrophil extracellular traps (NETs). This first line of defence by neutrophils is known as NETosis, and requires autophagic activity. Treatment of neutrophils with mTOR inhibitors WYE-354 or rapamycin led to an increased release of NETs upon stimulation of neutrophils with a bacterial-derived stimulant, formyl-Met-Leu-Phe (fMLP), and an elevation in autophagosome formation [68]. These studies suggest mTOR signalling plays an important role in neutrophil activation. However, a decrease in NET formation in neutrophils treated with LPS in the presence of mTOR inhibition has also been reported, due to reduced HIF1-α translation, therefore further clarification is required [69]. Inhibition of mTORC2 activity through *RICTOR* KD severely abrogates chemotaxis in neutrophils through the inhibition of polarisation and directed migration induced by chemoattractants. The chemoattractants are unable to generate cAMP, thereby perturbing the cAMP/RhoA signalling axis [70]. This pathway appears to be regulated by PKCβII (downstream from mTORC2) as *PRKCB* KD leads to a similar disruption in chemotaxis as with mTORC2 inhibition. There is a significant decrease in PKCβII expression with *RICTOR* KD which disrupts subsequent translocation of PKCβII to the membrane to bind chemoattractant-induced adenylyl cyclase 9 and activate chemotaxis [71].

Ablation of mTORC1, *in vitro* via mTOR inhibitor torin-1 or *in vivo* in myeloid-specific transgenic mouse models, revealed an increase in eosinophil differentiation in the presence of IL-5, a cytokine involved in the recruitment of eosinophils in the presence of allergy. This increase was partially related to an up-regulation of GATA1 with mTOR inhibition. Interestingly, mTOR deletion in myeloid cells promotes eosinophil development but disrupts neutrophil development [72].

### B lymphocytes

B cell development comprises several phenotypic stages enabling B cell lineage commitment/maturation from HSCs initially in the FL during embryogenesis and then in the BM. Recombination of heavy and light immunoglobulin (Ig) chain genes form a platform for B cell development: comprising the rearrangement of the variable (V), diversity (D) and joining (J) gene segments of the Ig heavy chain initially, followed by VJ rearrangement of the Ig light chain genes. B cells that generate functional rearrangements express a B cell receptor (BCR) and can be stimulated to produce antibodies that recognise specific antigens [73]. Immature B cells, expressing surface-bound IgM, migrate into the spleen where they mature into naïve, follicular or marginal zone (MZ) B cells via transitional phases (T1–T3). Follicular 1 cells (B220^+^IgD^lo^CD21^lo^IgM^hi^) form the bulk of the circulating population, whereas follicular 2 cells (B220^+^IgD^hi^CD21^hi^IgM^hi^) form one-third of the circulating population and are considered to be more primitive [74]. Further migration into the lymph nodes as memory B cells or plasma cells is also observed.

The importance of mTOR signalling during B cell development is evident from a study analysing mice in which mTOR expression is reduced [68]. A reduction in progenitor B cells was observed in these mice, with a block between large pre-B cells (B220^+^CD24^+^CD43^+^) and small pre-B cells (B220^+^CD24^+^CD43^−^). Within the spleen, an increased number of mature B cells (B220^+^IgD^high^CD21^+^IgM^−^) and decreased T1 and T2 transitional B cells were observed compared with wild-type mice [68]. Deletion of the TSC1 complex in B cells (*CD19*-Cre) renders mTOR constitutively active, resulting in a partial block in B cell maturation as indicated by an elevation in T1 and T2 transitional B cells, and a depletion of MZ B cells [75].

Assessing mTORC1 signals more directly, *Raptor* cKO mice exhibit a significant decrease in B cell generation, due to an early block in lineage commitment [60,76]. For this reason, the effect of *Raptor* ablation on B cells was analysed using B cell-specific models (*Mb1-*Cre), which resulted in a profound block at the pre-B cell stage abrogating B cell maturation, proliferation, germinal centre reaction and antibody production [77]. A cKO of *Raptor* specifically in B cells (hCD20-Tam-Cre) resulted in a decrease in germinal centre B cells and nascent antibody-secreting plasma cells and the elimination of germinal centres, resulting in a decline in serum antibodies [77]. These studies illustrate the importance of mTORC1 at multiple stages of B cell maturation, and highlights the critical role played by mTOR in mounting an appropriate humoral immune response.

*Rictor* KO models revealed a role for mTORC2 during B cell maturation, resulting in a decrease in mature B cells [78]. Studies demonstrate an increase in early B cell populations including the pro-B, pre-B and immature B cells in *Rictor* cKO mice, characterised by elevated FOXO1 and RAG1 expression and a subsequent reduction in mature splenic B cells [52]. However, HSCs isolated from *SIN1*^−/−^ mice reconstituted haemopoietic lineages, suggesting a minimal role of mTORC2 in B cell development during early stages. These mice exhibited increased IL7 production and RAG1/2 expression at the pro-B stage and fewer IgM^+^ immature B cells, suggesting a role for mTORC2 after B lineage commitment [79]. mTORC2 has also been shown to play an important role in B cell survival, as *Rictor*^−/−^ mice display increased caspase-3 and PARP expression, together with increased cell death and a decrease in B-cell activating factor (BAFF) expression in mature B cells [58]. Lee et al. proposed that mTORC2 regulates canonical and non-canonical NFκB signalling pathways responsible for mature B cell maintenance and survival [78]. Interestingly, *Rictor*^−/−^ mice possess increased CIP2A binding to PP2A, leading to increased c-myc phosphorylation and expression, and decreased E2F1 expression, which leads to apoptosis [80].

### T lymphocytes

T cells develop in the thymus, undergoing rigorous positive and negative selection processes at the CD4^+^CD8^+^ double positive (DP) stage of development, to generate a pool of T cells that recognise foreign peptides in the context of self MHC-I (CD8^+^-cytotoxic T cells; Tc) or MHC-II (CD4^+^-helper T cells; Th). Analysis of mTORC1 signalling inhibition during T cell development showed that rapamycin treatment and *Raptor* deletion in cKO mice resulted in reduced thymic cellularity and a decrease in the proportion of DP cells, coupled with a concomitant increase in CD4^−^CD8^−^ double negative (DN) cells [81]. Within the DN population, rapamycin blocked T cell development at the DN3 stage, probably prior to the proliferative burst associated with β-selection, while development was arrested at the DN1–DN2 transition in *Raptor*^−/−^ mice both *in vitro* and *in vivo*. This block was associated with a reduction in proliferation due to an instability of cyclinD/CDK6 complexes. Similar results were noted with an *mTOR* cKO mouse model, while *Rictor* cKO mice exhibited a block in proliferation at the DN3 stage of development [82,83], suggesting that mTORC1 plays a critical role, which is distinct from mTORC2, during the early stages of T cell development.

The thymic micro-environment plays a critical role in enabling the appropriate development of nascent T cells, particularly thymic epithelial cells (TECs). Selective *Rictor*^−/−^ in TECs results in a reduction in thymic mass and cellularity of TECs, and decreased generation of specific T cell lineages: TCRαβ, TCRγδ, invariant NKT [84] and regulatory T cells, thereby revealing an important role of mTORC2 in thymopoiesis and T cell lineage generation [85].

In the periphery, reduced mTOR expression/activity decreased T cell numbers, T cell activation and proliferation [68]. Furthermore, TSC1 ablation drove naïve T cells from quiescence to a poor immune response, altering the cell size and cycling [86]. mTORC1 activation, through TSC2 deletion, led to increased, terminally differentiated effector CD8^+^ T cell formation not capable of conforming to a memory T cell phenotype. However, mice deficient in mTORC1 activity through deletion of RHEB, led to loss of effector CD8^+^ T cell formation with no change in memory T cell expression, suggesting a role of mTORC1 in the differentiation of specific T cell subsets [87]. Indeed, mTORC1 is involved in Th1 and Th17 differentiation from naïve CD4^+^ T cells, as deletion of RHEB blocks Th1/Th17 differentiation, but not Th2 differentiation *in vivo* and *in vitro* [88]. mTORC1-mediated signalling plays a critical role in CD4^+^ T cell proliferation, by enhancing PPARγ activity, which in turn activates fatty acid metabolism, thus enabling the metabolic reprogramming required to activate CD4^+^ T cells [89].

Th cells require mTORC2 for differentiation as ablation of mTORC2 in T cells led to impaired Th1 and Th2 differentiation, which could be reversed by activating AKT and PKCθ, respectively [90]. However, mTORC2 is mainly considered to regulate Th2 differentiation, as *Rictor* KO in CD4^+^ T cells specifically led to the generation of Th1 and Th17 cells but not Th2 cells [91]. mTORC2 controls CD8^+^ T cell differentiation in a FOXO1-dependent manner as ablation of mTORC2 led to an increase in memory precursor effector cells and not in the short-lived effector cells driven by *Eomes* and *Tcf1* up-regulation caused by FOXO1 [85,92]. Recently, Velde and Murray demonstrated that mTORC2 plays an important role in micro-environment sensing in CD4^+^ T cells. In a normal setting, CD4^+^ T cells require essential and non-essential AAs to undergo cell division. Limiting arginine and leucine resulted in cell cycle disruption, which could be bypassed in the absence of *Rictor*. This resulted in cells initiating the cell cycle regardless of limiting AAs, thus bypassing micro-environmental sensing [93].

## Conclusion

Since the discovery of mTOR nearly 25 years ago, considerable data have been generated regarding its cellular functions. Through mTORC1 and mTORC2, the mTOR signalling pathway plays a critical role in protein and lipid biosynthesis, cell survival, migration, cell development and cell cycling. This is achieved through precise regulation of mTOR activity, which are tightly controlled through cross-talk with additional signalling pathways such as the ERK/MAPK and Wnt-signalling cascades. Here, we have highlighted the importance of mTOR in haemopoiesis, discussing the essential nature of mTORC1 during embryogenesis and haemopoiesis, regulating HSC self-renewal capacity, long-term potentiation and lineage commitment. On the other hand, genetic studies have revealed a more subtle role for mTORC2, establishing key roles in cell survival and later stages of haemopoietic cell lineage development, and supporting the haemopoietic niche. Further dissection of the physiological roles of mTOR as it resides in these complexes will provide fundamental information to assist in the development of therapeutic compounds to target specific mTOR functions.

## Abbreviations

AAs: amino acids
AMPK: AMP-protein kinase
BCR: B cell receptor
BM: bone marrow
BMDMs: BM-derived macrophages
cKO: conditional knockout
CLPs: common lymphoid progenitors
CMPs: common myeloid progenitors
DN: double negative
DP: double positive
FL: foetal liver
FOXO: Forkhead Box O
GF: growth factor
GSK3: glycogen synthase kinase 3
HSCs: haemopoietic stem cells
IMLECs: innate myelo-lymphoblastoid effector cells
KD: knockdown
KO: knockout
LSK: Lin^−^Sca1^+^cKit^+^
MPP: multipotent progenitor
mTOR: mechanistic target of rapamycin
MZ: marginal zone
NETs: neutrophil extracellular traps
PDK1: 3-phosphoinositide-dependent protein kinase 1
PI3K: phosphoinositide 3-kinase
PIP_2_: phosphatidylinositol-4,5-phosphate
PIP_3_: phosphatidylinositol-3,4,5-phosphate
PKCα: protein kinase C-alpha
PTEN: phosphatase and tensin homolog
RBC: red blood cell
RHEB: Ras homolog enriched in brain
ROS: reactive oxygen species
S6K1: S6 Kinase 1
SGK1: serum and glucocorticoid-induced protein kinase 1
TECs: thymic epithelial cells
TLR4: toll-like receptor 4
